# Baofukang suppository promotes the repair of vaginal epithelial cells in response to *Candida albicans*

**DOI:** 10.1186/s13568-016-0281-1

**Published:** 2016-11-09

**Authors:** Ting Li, Xiaoxi Niu, Xu Zhang, Suxia Wang, Zhaohui Liu

**Affiliations:** 1 Department of Obstetrics and Gynecology, Peking University First Hospital, 8 Xishiku Street, Xicheng District, Beijing, 100034 China; 2 Laboratory of Electron Microscopy, Ultrastructural Pathology Center, Peking University First Hospital, Beijing, 100034 China

**Keywords:** Vulvovaginal candidiasis, Vaginal epithelial cells, *Candida albicans*, Baofukang suppository, Cytokines

## Abstract

Vulvovaginal candidiasis (VVC) is an opportunistic fungal infection predominantly caused by *Candida albicans* affecting a significant number of women of reproductive age. The Chinese medicine, the Baofukang suppository is widely used in the clinic for its antimicrobial activity and is therefore of great interest as a potential antifungal drug for the prevention of VVC. We evaluated the cytotoxic activity of the Baofukang suppository using the VK2/E6E7 vaginal epithelial cell (VEC) line. When treated with the Baofukang suppository, all of the immunocompetent cytokines and chemokines (e.g., IL-2, IL-4, IL-6, IL-8, and IL-17) by infected VK2/E6E7 cells was statistically up-regulated (*P* < 0.05), except IL-4 (11.70 ± 1.82 vs. 14.88 ± 4.72, *P* = 0.343) compared to the infected control cells. The secretion of non-B IgG also exhibited the same trend. Our scanning electron microscopy results revealed that *C. albicans* can invade VECs by both induced endocytosis and active penetration. The Baofukang suppository could effectively inhibit the adhesion, hyphal formation, and proliferation, as well as notably restore the vaginal epithelial cell morphology, viability, and enhance the local immune function of the VECs. These preliminary results suggest promising antimicrobial properties of the Baofukang suppository, which may be efficacious as an antifungal therapy candidate via up-regulating Th1 cellular immunity, the Th17-axis of the innate immune response, and the secretion of vaginal epithelial-derived IgG. These combined effects collectively restore the immune function of the infected VECs against *C. albicans* in vitro.

## Introduction

The majority of women experience at least one episode of vulvovaginal candidiasis (VVC) in their lifetime (Sobel et al. 1998). The vaginal epithelium as a mucosal surface is of immense importance in host defense and immune surveillance (Moyes et al. 2011). Specifically, it functions as the first line of host defense against pathogen invasion to provide a physical barrier and protect underlying tissues and organs (Cole 2006).

Products containing essential oils have been formulated for intra-vaginal use, and are often recommended as home remedies for the treatment of vaginal candidiasis by published books and articles (Marti and Hine 1995; Newall et al. 1996). Baofukang suppository is a type of traditional Chinese medicine that consists primarily of two Chinese Medicinal Herbs: (1) zedoary turmeric oil; and (2) borneol. Zedoary turmeric oil is a volatile oil steam-distilled from Curcuma phaeocaulis which contains a variety of effective components, including beta-elemene, curcumin, curzerenone, and Zimmer ketones currently in clinical use. These components may provide potent pharmacological activities, such as antitumor or antiviral effects, enhancing the immune response, particularly by promoting the regeneration and repair of damaged or inflamed mucosa (Kamazeri et al. 2012). Due to its potent pharmacological action, this plant extract has begun to attract some significant attention. Borneol is extracted from the essential oil of various subtropical and evergreen broad-leaved such as Cinnamomum and Lauraceae, and a bicyclic monoterpenoid alcohol commonly used in traditional Chinese medicine as the adjuvant for more than 1500 years (Jiang et al. 2008). It demonstrates anti-inflammatory, analgesic, and antibacterial properties, while also accelerating percutaneous drug absorption and increasing the bioavailability of drugs in the brain tissue (Almeida et al. 2013; Slamenova et al. 2009). Therefore, the aims of this work was to evaluate the in vitro antifungal properties and mechanisms of the Baofukang suppository, which would be further applied widely in clinic as a potential antifungal drug for the prevention of VVC.

## Materials and methods

### Vaginal epithelial cell culture

The VK2/E6E7 vaginal epithelial cell line (ATCC® CRL-2616), obtained from the American Type Culture Collection (ATCC; Rock ville, MD, USA) is an epithelial cell line derived from the vaginal mucosa of a healthy premenopausal female undergoing vaginal repair surgery, that was subsequently immortalized with human papillomavirus 16/E6E7. VK2 cells were cultured in keratinocyte-serum free medium (K-SFM, Gibco, USA) supplemented with 5 ng/mL recombinant epidermal growth factor and 50 µg/mL bovine pituitary extract (Invitrogen Corporation, Grand Island, NY, USA), 100 U/mL penicillin (Life Technologies, Grand Island, NY, USA), 100 µg/mL streptomycin (Life Technologies) and 0.4 mM CaCl_2_ at 37 °C with 5% CO_2_ and a high humidity environment. A subculture of the cells was performed every 3–4 days.

### Microbial strains and growth conditions

*Candida albicans* collection strains (ATCC-11006) were grown aerobically overnight on Sabouraud-dextrose agar plates (Becton Dickinson, Cockeysville, MD, USA) at 37 °C until the mid-exponential growth phase. The blastoconidia were collected and resuspended in RPMI 1640 and adjusted to 1.0 × 10^5^ cells/mL after counting with a hemocytometer (Hausser Scientific; Horsham, PA, USA).

### Drug preparation

Baofukang suppository (Hainan bikai Pharmaceutical Co., Ltd.) is a traditional Chinese Medicine, with every 1.74 g tablet consisting of 88 mg zedoary turmeric oil, 75 mg borneol, and other components as a preservation matrix. One vaginal suppository tablet (water soluble) was dissolved in 44 mL serum-free RPMI1640 culture medium to prepare a drug stock solution of 3.95 × 10^4^ µg/mL, and was passed through a 0.22 µM membrane filter for sterilization. All drug solutions were stored at −20 °C until further experiments.

### Evaluation of cytotoxic activity

CCK-8 (Dojindo Laboratories, Tokyo, Japan) was used to evaluate the cytotoxicity of the Baofukang suppository at a concentration of 5, 10, 20, 40, 80, and 160 µg/mL. A total volume of 200 µL VK2 cell suspension was seeded into each well of a 96-well microtiter plate and placed into a humidified atmosphere containing 5% CO_2_ at 37 °C for 24 h before the cells were treated. Untreated cells that received only media were used as the negative control. Concentrations of 0, 5, 10, 20, 40, 80, and 160 µg/mL Baofukang suppository were added to the VK2 cells for 24 h. The cells in each well were incubated in 100 µL K-SFM containing 10 µL CCK-8 reagents at 37 °C for 1 h. The plate was then shaken on an automatic mixer for 3 min and the absorbance at 450 nm (A_450_) was measured using a Multiscan GO micro-plate reader. The results were expressed as the percentage of cell viability and plotted.

$${\text{Cell viability }}\left( \% \right) = \left[ {{\text{A}}_{450} \left( {{\text{treated}}} \right) - {\text{ A}}_{450} \left( {{\text{blank}}} \right)} \right]/\left[ {{\text{ A}}_{450} \left( {{\text{control}}} \right) - {\text{ A}}_{450} \left( {{\text{blank}}} \right)} \right] \times 100\%$$

The concentration of the sample that inhibited 10% cell growth as calculated by SPSS 13.0 was the 10% inhibition concentration (IC_10_). This dose was defined as a safe dose with little poisonous side effects—the highest concentration where still no effect of the Baofukang suppository on cell viability (≥90% survival) (Namiecinski et al. 2004; Qiao et al. 2013).

### Cytokine and chemokine analysis of coculture supernatants

For the examination of cytokines and chemokines, epithelial cells (1 × 10 cells/mL) were cocultured with *C. candida* (1 × 10^5^/mL) at a ratio of 1:1 in separate wells for 12 h for the VK2 cell line cells in a total volume of 2 mL K-SFM complete medium in 24-well tissue culture plates (Costar, Corning, NY, USA). Following a coculture for 12 h, the culture medium was aspirated, washed three times with PBS, and replaced with 1 mL different concentrations 20 µg/mL of Baofukang suppository (IC_10_) as described above for additional 24 h. The supernatants were collected and centrifuged at 12,000*g* for 5 min and finally stored at −80 °C until an enzyme-linked immunosorbent assay (ELISA, eBioscience, USA) was performed. The supernatants were assayed for the levels of IL-6, IL-2, IL-4, IL-8, and IL-17 cytokines according to the manufacturer’s instructions. New standard curves were generated for every set of experiments. The absorbance values and concentrations of each cytokine were determined using a Ceres 900 automated microplate reader (Bio-Tek Corp., Wisnooski, VT, USA) and Kineticalc software (Bio-Tek). Each independent experiment was performed in triplicate.

### Epithelial-derived IgG and sIgA analysis of coculture supernatants

To further explore the local immune function of vaginal epithelial cells, we stimulated VK2/E6E7 with *C. candida* (1 × 10^5^/mL) and detected the level of secreted non-B IgG and IgA in the culture supernatants (collected as described above) by an ELISA (eBioscience). The ELISAs were conducted as mentioned above.

### Scanning electron microscopy (SEM)

Specimens were fixed overnight in 2.5% glutaraldehyde in 0.1 M sodium cacodylate buffer (pH 7.4; Electron Microscopy Sciences, Hatfield, PA, USA) at 4 °C, rinsed three times with PBS, dehydrated in graded ethanol (25, 50, 75, 95, and 100%), and dried using the critical point drying method (BALTEC, Balzers, Liechtenstein). The dried samples were glued onto SEM stubs, sputter-coated with a 10 nm thick layer of gold (BALTEC, Balzers, Liechtenstein), and examined using a scanning electron microscope (S-3400 N, Hitachi, Japan).

### Statistical analysis

All data are presented as the mean ± standard deviation of three independent measurements. The statistical analyses were conducted using SPSS version 13.0 (SPSS, Chicago, IL, USA). A statistical comparison between the groups was carried out using a one-way ANOVA. Subsequent comparisons were performed using the LSD method. Significant differences were defined as having a *P* value of less than 0.05.

## Results

### Effects of the Baofukang suppository on VK2/E6E7 cell viability

**Fig. 1:**
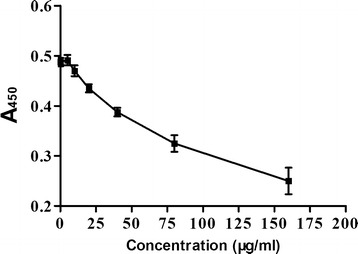
Cytotoxicity of VECs in the presence of the Baofukang suppository. The vaginal epithelial cells were treated with the Baofukang suppository for 24 h. The mean values for the remaining four values and standard deviations (*error bars*) are shown

To mimic clinical situations in which antibiotics or antifungals may be safe and well-tolerated in the human body, we determined whether the Baofukang suppository affects cell viability. The A450 of the VK2/E6E7 cells treated with the Baofukang suppository at doses of 0, 5, 10, 20, 40, 80, and 160 µg/mL were plotted in Fig. 1. As shown in Fig. 1, high doses (>20 µg/mL) of the Baofukang suppository did inhibit vaginal epithelial cell viability or growth, while there were no observed changes in the cells exposed to the low doses (≤20 µg/mL). The IC_10_ calculated by SPSS 13.0 was 19.89 µg/mL, and was considered to be a safe dose for VK2 cells with minimal toxic side effects, and was selected for further experiments.

### Baofukang suppository modulates cytokine release by human vaginal epithelial cells

**Fig. 2:**
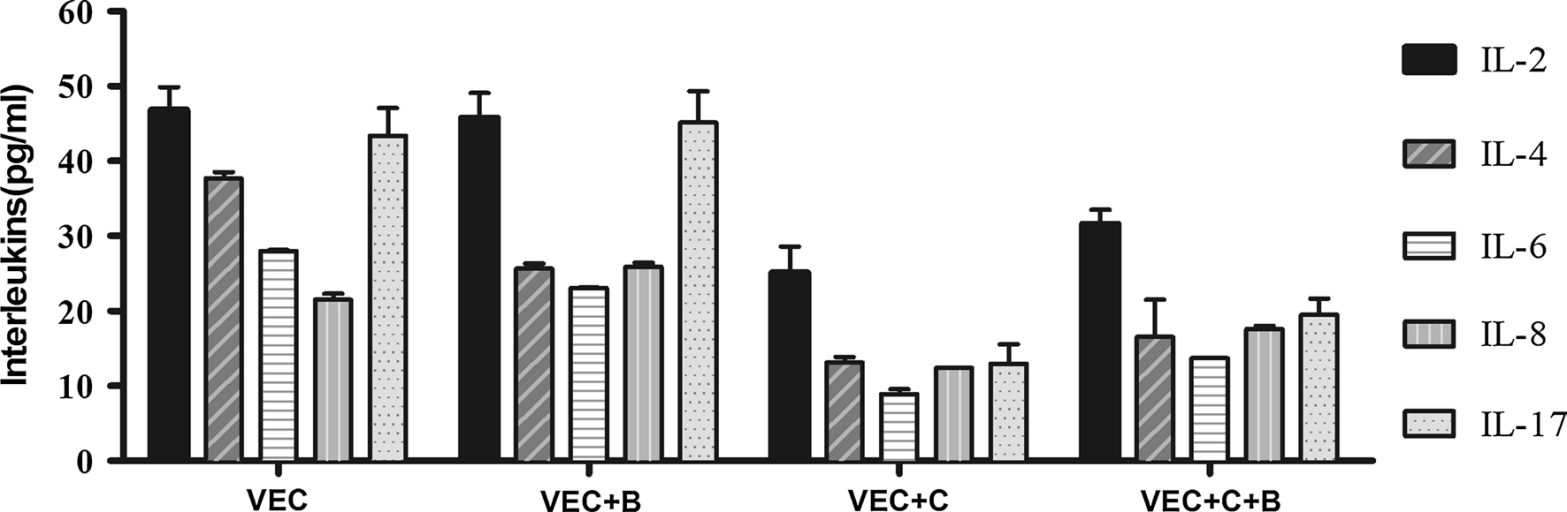
The production of IL-2, IL-4, IL-6, IL-8, and IL-17 (expressed as pg/mL) by the vaginal epithelial cell line, VK2/E6E7 cells cultivated alone, grown with 20 µg/mL the Baofukang suppository, and infected with *C. albicans* (1 × 10^5^/mL). The cytokine levels were measured after 12 h of infection and subsequent 24 h of co-incubation with 20 µg/mL of the Baofukang suppository. V represents the vaginal epithelial cells cultivated alone; V+B represents vaginal epithelial cells co-incubated with 20 µg/mL of the Baofukang suppository for 24 h; V+C represents vaginal epithelial cells infected with *C. albicans* for 12 h; V+C+F50 represents the vaginal epithelial cells infected with *C. albicans* for 12 h, then treated with 20 µg/mL of Baofukang suppository for another 24 h

In the present study, at baseline, the level of IL-2, IL-4, IL-6, IL-8, and IL-17 production by VK2 cells were 46.81 ± 3.07, 34.42 ± 5.60, 27.96 ± 0.15, 21.53 ± 0.78, and 43.36 ± 3.68 pg/mL, respectively (Fig. 2). After 24 h of co-incubation with 20 µg/mL of the Baofukang suppository alone, there were no significant changes in the levels of IL-2 (45.76 ± 3.31 pg/mL, *P* = 0.679) or IL-17 (45.13 ± 4.718 pg/mL, *P* = 0.526). Comparatively, the Baofukang suppository stimulated a significant up-regulation in the production of IL-8 (25.91 ± 0.50 pg/mL, *P* < 0.0001) and a significant down-regulation in the production of IL-4 (25.06 ± 1.65 pg/mL, *P* = 0.018) and IL-6 (23.08 ± 0.12 pg/mL, *P* < 0.0001) by the VK2/E6E7 cells was observed (Fig. 2).

After 12 h of challenge with *C. albicans*, IL-2, IL-4, IL-6, IL-8, and IL-17 had substantially declined (0.4- to 0.8-fold, *P* < 0.05; Fig. 2); however, when treated with 20 µg/mL Baofukang suppository, all of the above-mentioned cytokines in the infected VK2/E6E7 cell cultures were significantly up-regulated (IL-2: 25.14 ± 3.43 vs. 31.59 ± 1.90 pg/mL, *P* = 0.030; IL-4: 11.70 ± 1.82 vs. 14.88 ± 4.72 pg/mL, *P* = 0.343; IL-6: 8.91 ± 0.65 vs. 13.74 ± 0.51 pg/mL, *P* < 0.0001; IL-8: 12.41 ± 0.06 vs. 17.63 ± 0.41 pg/mL, *P* < 0.0001; IL-17: 12.99 ± 2.57 vs. 19.52 ± 2.13 pg/mL, *P* = 0.039; respectively), when compared to the untreated vaginal cells infected with *C. albicans* (Fig. 2). We determined the Th1/Th2 balance by calculating the IL-2/IL-4 ratio (Table 1).

**Table 1:** Th1/Th2 cytokines/ratios

Cytokine (pg/ml)	V	V+B	V+C	V+C+B	*F* value	*P* value
IL-2	46.81 ± 3.07	45.76 ± 3.31	25.14 ± 3.43	31.58 ± 1.90	38.291	<0.0001*
IL-4	37.65 ± 0.85	25.70 ± 0.60	13.12 ± 0.76	16.57 ± 4.99	54.464	<0.0001*
IL2/IL-4	1.24 ± 0.08	1.78 ± 0.17	1.93 ± 0.33	2.03 ± 0.63	2.684	0.117

V represents the vaginal epithelial cells cultivated alone; V+B represents the vaginal epithelial cells co-incubated with 20 µg/mL of the Baofukang suppository for 24 h; V+C represents the vaginal epithelial cells infected with *C. albicans* for 12 h; V+C+B represents the vaginal epithelial cells infected with *C. albicans* for 12 h, then treated with 20 µg/mL of the Baofukang suppository for another 24 h

* *P* < 0.0001

### The Baofukang suppository modulates epithelial-derived IgG secreted by human vaginal epithelial cells

**Fig. 3:**
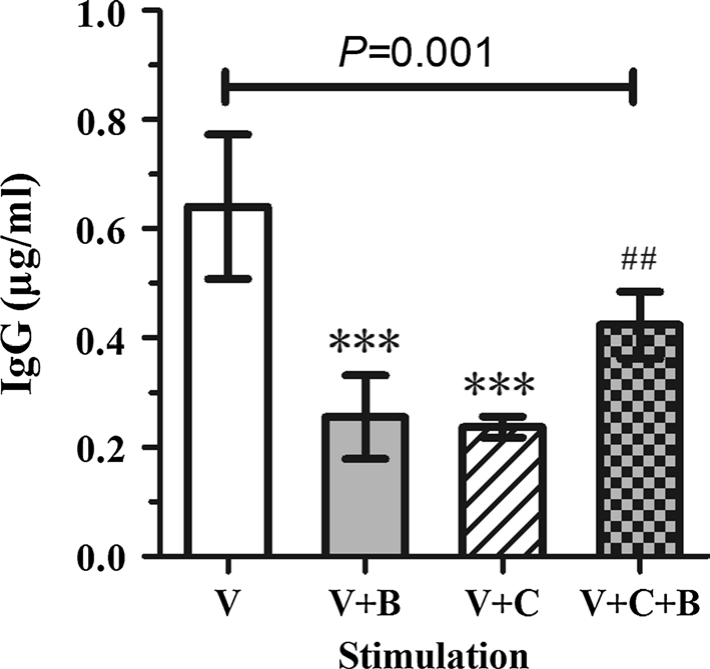
The production of epithelial-derived IgG (expressed in µg/mL) by the vaginal epithelial cell line, VK2/E6E7 cells. The IgG levels were measured after 12 h of infection and the subsequent 24 h of co-incubation with the Baofukang suppository. V represents the vaginal epithelial cells cultivated alone; V+B represents the vaginal epithelial cells co-incubated with 20 µg/mL of teh Baofukang suppository for 24 h; V+C represents the vaginal epithelial cells infected with *C. albicans* for 12 h; V+C+B represents the vaginal epithelial cells infected with *C. albicans* for 12 h, then treated with 20 µg/mL of the Baofukang suppository for another 24 h. ***Significant difference compared to the V group (*P* < 0.0001), ^##^significant difference compared to the V+C group (*P* = 0.025). Each sample was repeated three times. The *error bars* indicate the standard deviation

To determine whether infected, uninfected or treated vaginal epithelial cells secrete epithelial-derived IgG and sIgA, the culture supernatants were examined by an ELISA (Fig. 3). Secretory IgA (sIgA) that we previously assumed to be the most abundant Ig isotype secreted in culture supernatants was undetectable in this experiment (data not shown).

Surprisingly, we found that the VECs spontaneously secrete epithelial-derived IgG under baseline conditions (Fig. 3). The baseline level of IgG secreted by the VK2 cells was 0.64 ± 0.13 µg/mL, which dropped sharply following infection or co-incubation with the Baofukang suppository alone (*P* < 0.0001). However, when the infected VECs were treated with 20 µg/mL Baofukang suppository, the interaction pattern of this suppository was changed, and the level of IgG secreted by the treated VK2/E6E7 cells was statistically up-regulated (0.42 ± 0.06 µg/mL) over the untreated infected cells (0.24 ± 0.02 µg/mL) (*P* = 0.025). Despite this increase, the levels did not reach the baseline values (*P* = 0.013).

### Baofukang suppository promotes the repair of infected cells

For further insight into the interactions of *C. albicans* with VECs, VEC monolayers were preferred even though the vaginal mucosa is composed of stratified squamous epithelium because only the superficial epithelial cells that are exposed to the luminal surface interact with *C. albicans*. Thus, establishing models with multiple layers of cells could complicate the quantification of the interactions between the epithelial cells and the pathogen.

**Fig. 4:**
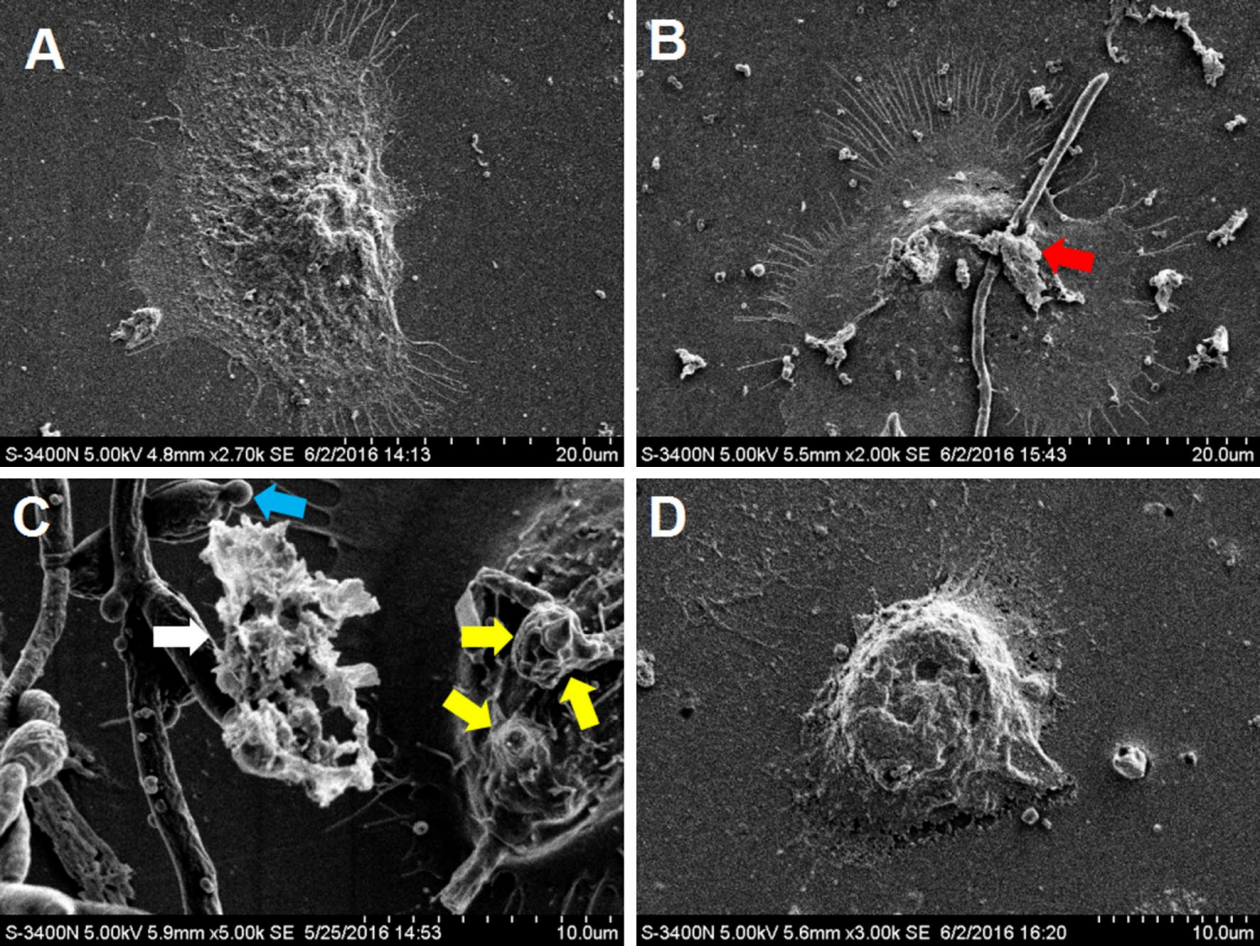
Scanning electron micrographs (SEM) of the vaginal epithelial cells. SEM of the control cells (**A**), *C. albicans* infected cells at 6 h (**B**), *C. albicans* infected cells at 12 h (**C**) and treated cells (**D**), the *latter* represents the vaginal epithelial cells infected with *C. albicans* for 12 h, then treated with 20 µg/mL Baofukang suppository for another 24 h. Fusion of the microvilli-like structures forming membrane leaflets that envelop the invading hyphal cells is indicated by the *red arrow*; damaged cell debris is indicated by the *white arrow*; pseudohyphae elements “engulfed” into vaginal epithelial cells are indicated by *yellow arrows*; a budding spore is indicated by *blue arrows*

The cell surface of the normal, uninfected cells was covered with microvilli or a microvilli crest that was an irregular, loose net-like membrane ruffle (Fig. 4A). At 6 and 12 h post-infection, epithelial cell adherence and invasion were observed (Fig. 4B, D). Our results suggest that *C. albicans* can invade vaginal epithelial cells by two distinct mechanisms that ultimately result in cellular damage. The first is the induction of cellular endocytosis by the *C. albicans* hypha, and the other is via active penetration without pseudopod formation and endocytosis.

After 24 h of treatment with the Baofukang suppository, as shown at ×3000 high magnification in Fig. 4D, the invasive blastoconidia and hyphae initially observed were significantly reduced or completely absent. Treated cells with a normal shape, good cell viability, relatively intact and smooth cell membrane, were well adhered to the wall and completely stretched, similar to the uninfected cells shown in Fig. 4A.

These results illustrate that the Baofukang suppository could not only effectively inhibit the adhesion, hyphal formation, and proliferation of *C. albicans*, but also notably restore the VEC morphology and viability. Thus, this serves to enhance the local immune function of the VECs.

## Discussion

VECs lining the mucosal surfaces of the vagina provide a first-line barrier defense system for both innate and acquired immune functions. As in anticandidal mucosal immunity, the innate role of the epithelial response involves the release of epithelial cell-derived cytokines and chemokines in response to *C. albicans* appear to have important roles recruiting and activating a variety of immune cells, immunoregulation, and tissue repair (Dongari-Bagtzoglou et al. 2005; Schaller et al. 2004). This serves to further mediate the innate and adaptive responses of protective mucosal immunity against *C. albicans*. Our results have demonstrated that VECs secrete various soluble immunological mediators under normal culture conditions. However, after challenging the cells with *C. albicans* for 12 h, the secretion of all the cytokines and chemokines produced by the VECs were significantly decreased. This decrease may partially be due to a direct invasion of *C. albicans* and a strongly diminished total number of VK2/E6E7, resulting in damaging the local innate immune function. Martinez et al. (2009) confirmed that the numbers of VK2/E6E7 cells remained stable within 6 h of *C. albicans* infection, which decreased to 1/8-fold after inoculation for 12 h.

To mimic the effect of the Baofukang suppository in treating vulvovaginal candidiasis under in vivo conditions, the VK2/E6E7 cells were infected with *C. albicans* for 12 h and then treated with the Baofukang suppository for 24 h. The levels of IL-2, IL-6, IL-8, and IL-17 were significantly increased, while IL-4 was only slightly increased in the treated cells compared with the infected control. Th1 cells are associated with interferon γ or L-2 cooperating with cytotoxic CD8+ T cells, while Th2 cells are associated with IL-4 or IL-6 promoting a humoral, proinflammatory response (Schilling et al. 2007; Woo et al. 2015). A increase in the balance of the Th1/Th2 ratio is associated with the enhancement of cell-mediated immunity. Thus, the Baofukang suppository may enhance or restore a protective Th1 response in infected cells, which represents the immunological hallmark of candidal lesions believed to play a crucial role in the clearance of mycotic infection (Fidel 2002). IL-2 and IL-4 are the classically representative of Th1 and Th2 cytokines, respectively (Fidel and Sobel 1994; Romani 1999; Woo et al. 2015). A strong role for Th1-type cell-mediated immunity against Candida was demonstrated by various experimental models (Klein et al. 1984; Clift 1984). An increased Th1/Th2 ratio (IL-2/IL-4) is associated with the activation of cell-mediated immunity and would be potentially beneficial for pathogen or cancer elimination, while a decreased ratio would raise the risk of inflammation progression. Our results indicate that the IL-2/IL-4 ratio was significantly increased in the Baofukang suppository-treated cells and indicates that the Baofukang suppository can indirectly up-regulate the vaginal local cellular immunity under the infective status promoting the host defense against invading pathogenic microorganisms.

Moreover, evidence has been mounting that a Th17-driven immune response plays a predominant role in modulating a defensive mucosal immune response against Candida in both mice and humans (Huang et al. 2004; Conti et al. 2009; Eyerich et al. 2008). An impaired IL-17 response appears to be responsible for the pathogenesis of chronic mucocutaneous candidiasis (Eyerich et al. 2008). Our data also affirm that the Baofukang suppository partly restores IL-17-production by VECs against Candida infection in vitro, thus, initiating an early Th17-type innate immune response against extracellular Candida adhesion and filamentation. Active cytokine production by VECs would enable a rapid response to an infection and provide protection within the vaginal microenvironment.

Immunoglobulins are one of the key molecules of the humoral immune response, and they have previously thought to be produced only by B cells, and no other cell types. However, 20 years ago, a series of studies demonstrated that non-B cancer cells and normal non-B cells (Qiu et al. 2003, 2013; Zhao et al. 2011) are capable of producing Igs, but it remains unclear whether normal VECs express functional Ig molecules. We cultured the immortalized VEC lines, VK2 cells, and used an ELISA to determine whether IgG or sIgA was secreted by the VECs. To our surprise, sIgA was undetectable in cell supernatants. In this report, we first noted epithelial-derived IgG were secreted by VECs in vitro, which challenges the classical concept that B cells are the only source of Ig. Antibody-mediated protection containing an anti-secreted aspartyl proteinase antibody or anti-Candida-mannoprotein appear to play an indispensable role in mucosal immunity against vaginitis. However, this hypothesis cannot explain the clinical phenomenon that no antibody deficiency was observed in recurrent VVC women and the absence of protective Candida-specific antibody production in inoculated mice (Ashman et al. 2004). Our study confirms our hypothesis that non-B IgG can be expressed in VECs, and they appear to be involved in the innate immune response of the vagina against mycotic infections, which can be partially repaired by the Baofukang suppository treatment. Further studies should ascertain if vaginal epithelial-derived IgG participates in the local mucosal immunity of the vagina against various common pathogens.

The SEM observations were first conducted to visualize the different stages (6 and 12 h) of the interaction of *C. albicans* with VECs. Similar to the pathological processes of vulvovaginal candidiasis, the yeast and filamentous forms of *C. albicans* are capable of adhering and invading the VECs, enabling the fungal cells to translocate across the vaginal mucosal barrier. *C. albicans* invade the vaginal epithelial monolayer via two mechanisms: (1) induced endocytosis; or (2) active penetration (Zhu and Filler 2010; Dalle et al. 2010; Naglik et al. 2011). Endocytosis is defined as the engulfment of epithelial cells with membrane protrusions at the point of entry of the invading hypha, and active penetration is defined as hypha penetration on the epithelial cell surface at their apical side at the point of entry of an invading hypha (Dalle et al. 2010). The two mechanisms appear throughout the entire process of invasion, while the former occurs more frequently during the early-stage and the latter more frequently in the late-stage in vitro (Dalle et al. 2010). We also confirm that hypha formation is the key to fungal invasion and damage, and VK2 cells, when not fully damaged by fungal cells, can kill or “engulf” the attached *C. albicans*. However, this antifungal phenomenon was not observed when the VK2 cells were killed by *C. albicans*.

When treated with a Baofukang suppository for 24 h compared with an infectious condition, *C. albicans* adhesion, invasion, and cellular injury could be restored to a normal condition. The antimicrobial activity of the Baofukang suppository of *C. albicans* could, in part, be associated with a major constituent of Zedoary turmeric oil 6. Moreover, these results also demonstrated a direct role for the Baofukang suppository in inhibiting germ tube formation and hyphal elongation for antifungal defense at mucosal surfaces. Therefore, it can be used as a natural preservative in food or pharmaceuticals.

In summary, the Baofukang suppository could not only effectively inhibit the adhesion, hyphal formation, and proliferation of *C. albicans,* but also notably restores the VEC morphology and viability, thereby enhancing the local immune function of these cells. The preliminary results suggest promising antimicrobial properties of the Baofukang suppository, which may be efficacious as an antifungal therapy via up-regulating Th1-type cellular immunity, the Th17-axis, and the secretion of vaginal epithelial-derived IgG. These responses serve to restore the immune function of the infected VECs against *C. albicans* in vitro.

## Abbreviations

VVC: vulvovaginal candidiasis
ATCC: American Type Culture Collection
K-SFM: keratinocyte-serum free medium
IC_10_: the 10% inhibition concentration
ELISA: enzyme-linked immunosorbent assay
SEM: scanning electron microscopy
sIgA: secretory IgA
VECs: vaginal epithelial cells
